# The paradox of eGFR trends and kidney failure incidence in patients without monogenic kidney disorders. Reply.

**DOI:** 10.1172/JCI187783

**Published:** 2024-12-16

**Authors:** Mark Elliott, Krzysztof Kiryluk, Ali Gharavi

**Affiliations:** 1 Department of Medicine, University of British Columbia, Vancouver, British Columbia, Canada.; 2 Columbia University, New York, New York, USA.

**Keywords:** Genetics, Nephrology, Genetic variation

**The authors reply:** Dr. Wang, thank you for the recognition of the important clinical implications of this work. In regard to the issue of CureGN participants with diabetes, the exclusion of individuals with diabetes occurred at the time of enrollment into the study, not at the time of biopsy. We used the presence of diabetes mellitus at the time of kidney biopsy as a covariate in our analyses, as diabetes is well described to influence kidney disease progression and outcomes (1). Only a small proportion of the CureGN cohort (18 individuals, 1%) had diabetes mellitus at the time of biopsy that had resolved at enrollment and were thus included in the analysis cohort.

With regard to the apparently discordant eGFR slope and kidney failure risk in the CureGN cohort without monogenic kidney disorders, this is likely due to the impact of treatment and remission rates on eGFR and kidney failure. As demonstrated in multiple glomerular disorders, including IgA nephropathy and focal segmental glomerulosclerosis, remission is a major driver of clinical outcomes including kidney failure risk and eGFR decline (2–4). A prior study of the CureGN IgA nephropathy cohort demonstrated that there is an improvement in eGFR between biopsy (mean 70.6 mL/min/1.73 m^2^) and enrollment (mean 75.8 mL/min/1.73 m^2^) as well as (5) focal segmental glomerulosclerosis, membranous nephropathy, or IgA nephropathy (IgAN). Within the CureGN cohort without monogenic kidney disorders, 67% (1,144 individuals) achieved complete remission within the follow-up time. Within this group who achieved complete remission, 4% (45 individuals) reached kidney failure and overall these individuals experienced an eGFR increase of 0.82 mL/min/year. Within the group who did not achieve complete remission, 23% (127 individuals) reached kidney failure and overall these individuals experienced an eGFR decline of 2.68 mL/min/year (*P* value for difference in eGFR slope < 0.001; Figure 1). Thus there is a group of individuals who continue to have progressive kidney disease and develop kidney failure over time, mainly those who do not achieve complete remission, while the majority of the cohort experiences an improvement in their eGFR with a lower risk of kidney failure driving the average eGFR change upwards and a lower risk of kidney failure overall. These findings are well aligned with the literature and highlight the observation that individuals with monogenic kidney disorders are less likely to achieve complete remission.

## Figures and Tables

**Figure 1:**
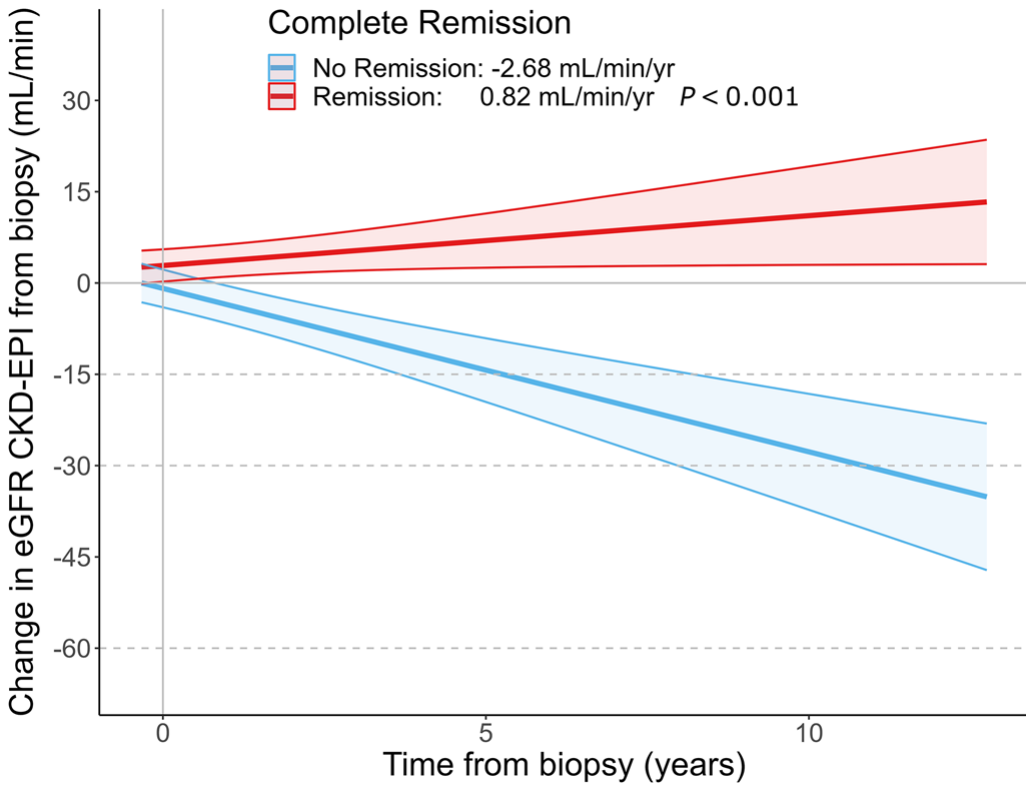
eGFR decline in the CureGN group without monogenic kidney disorders evaluated by achievement of complete remission, reported as mean with 95% CI displayed. CKD-EPI, chronic kidney disease epidemiology collaboration.

## Version 1 12/16/2024

Electronic publication
